# Supplementary material for: A bispecific antibody exhibits broad neutralization against SARS-CoV-2 Omicron variants XBB.1.16, BQ.1.1 and sarbecoviruses
Source: Nat Commun. 2024 Jun 15;15:5127. doi: 10.1038/s41467-024-49096-1 (PMC11180174; doi:10.1038/s41467-024-49096-1)
Supplement: Supplementary file 1 — Supplementary Information [file 41467_2024_49096_MOESM1_ESM.pdf]

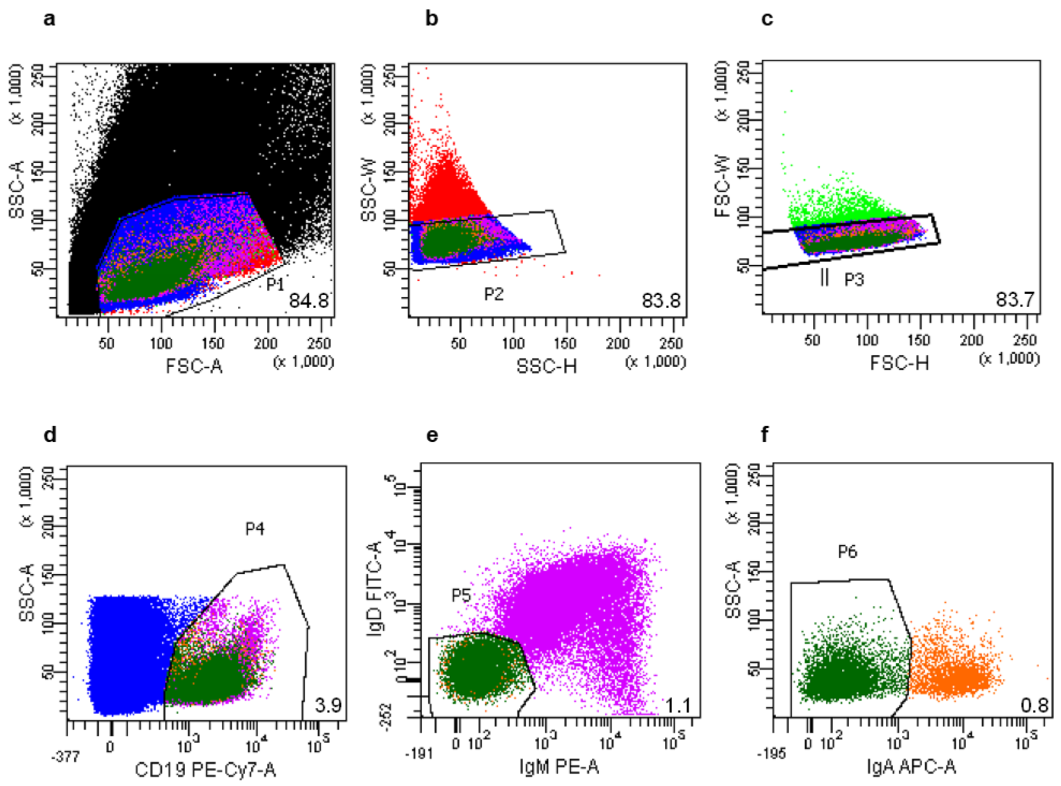

Supplementary Fig. S1. The gating strategy for the isolation of the memory B cell subset (CD19+IgA−IgD−IgM−) from PBMCs involved a multi-step process using flow cytometry. The following steps were employed: Initially, lymphocytes were identified and gated from the overall PBMCs (**a**). Within the lymphocytes gate, single memory B cells were further identified and gated (**b**, **c**, **d**). Within the single memory B cell gate, further gating was applied to exclude B cells expressing IgD (**e**), IgM (**e**), and IgA (**f**). The IgG-positive memory B cells were selectively chosen by excluding the other Ig isotypes. The numbers in the gates represent the percentage of cells relative to the total number of cells. Flow cytometry data were collected by BD BD FACSAria II and analyzed by FlowJo_v10.

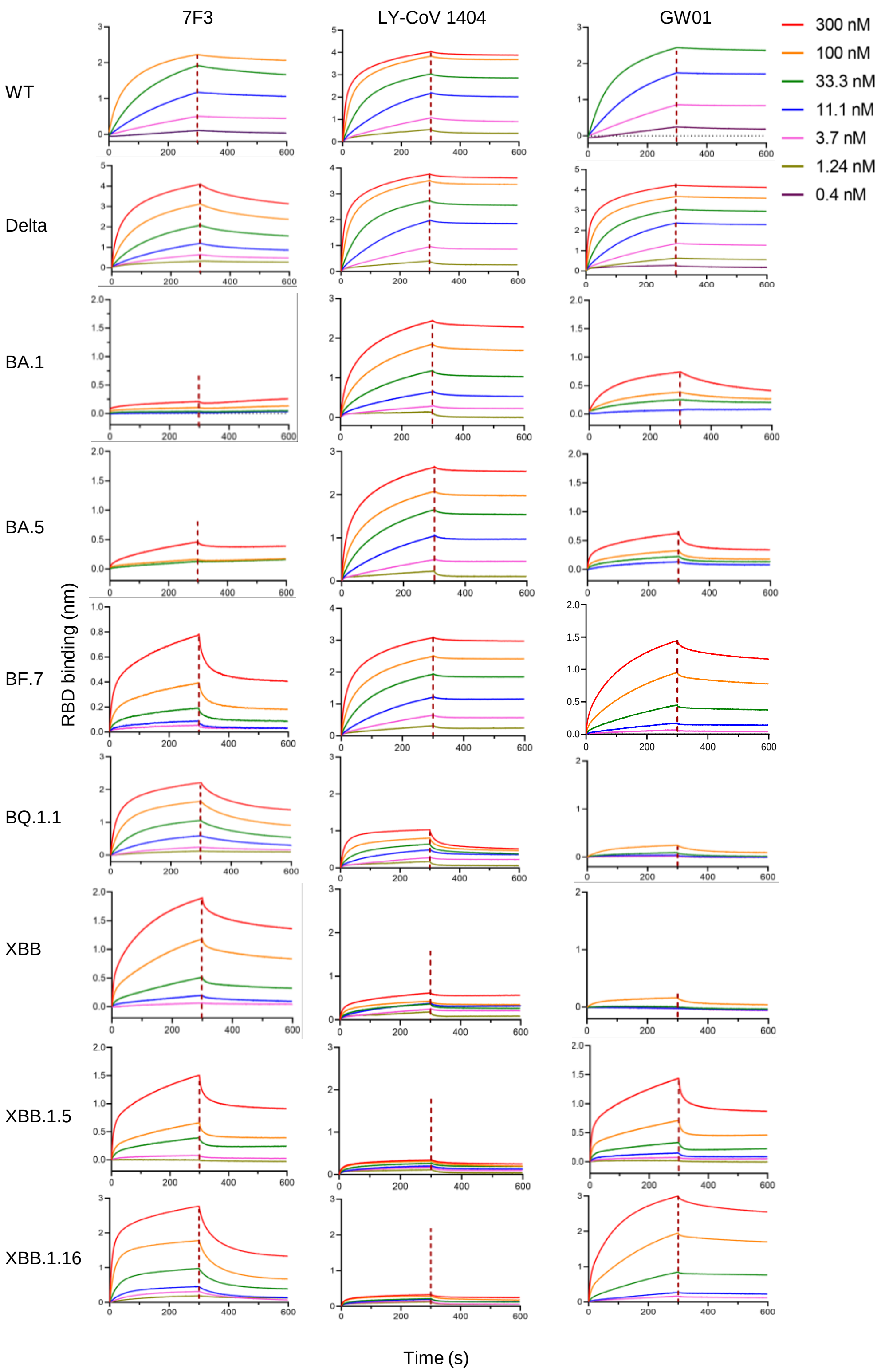

Supplementary Fig. S2. The association and dissociation curves of 7F3, LY-CoV1404, and GW01 to the RBDs from different variants as measured by BLI. These curves were crucial for determining the binding constant (KD). The experiments

were performed with a sequential 3-fold dilution of antibodies, commencing from 300 nM and progressing to 0.4 nM. Source data are provided as a Source Data file.

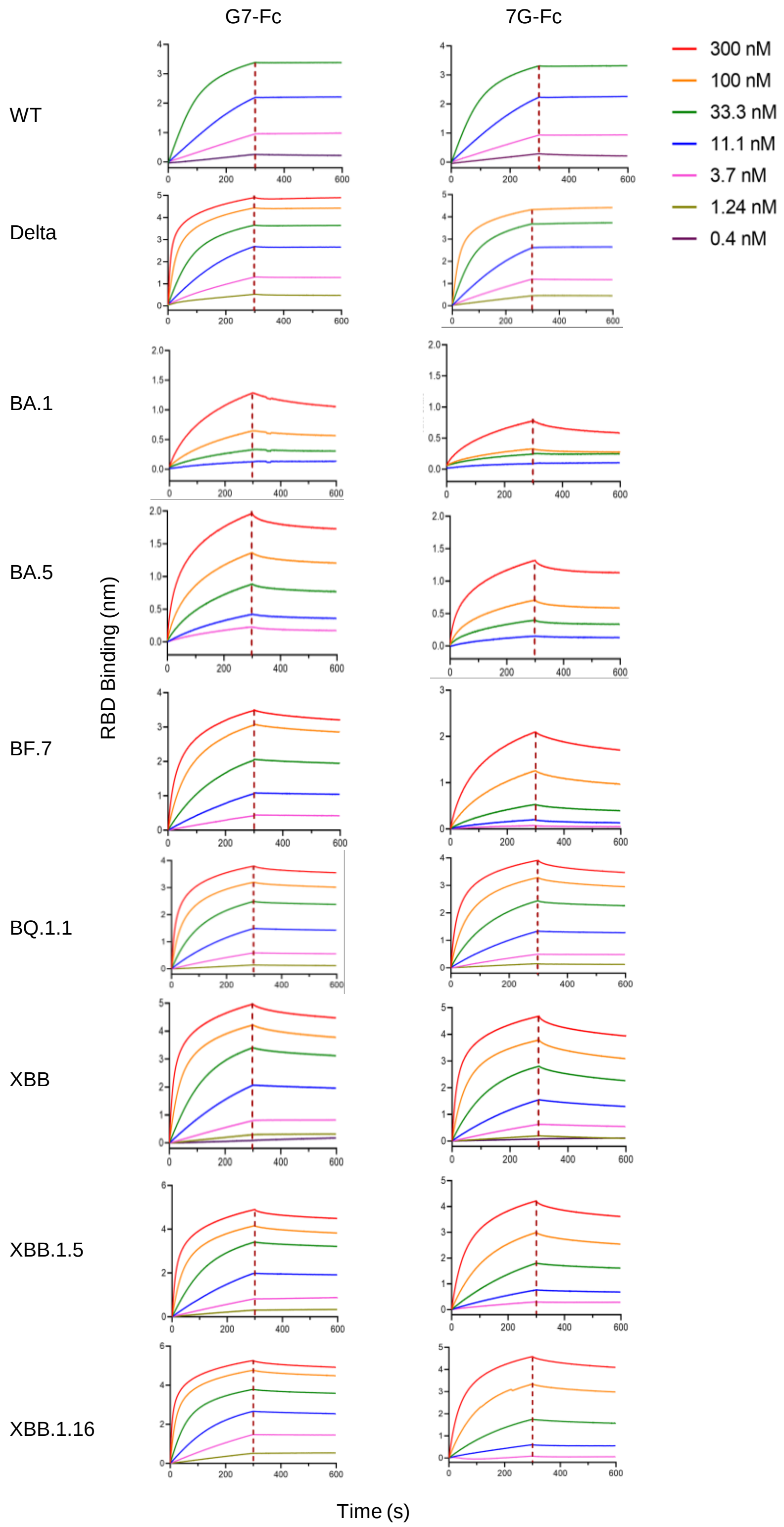

Supplementary Fig. S3. The association and dissociation curves of G7-Fc and 7G-Fc with RBDs from different variants, as measured by BLI. The experiments involved a stepwise 3-fold dilution of the antibody, ranging from 300 nM (red) to 0.4 nM (purple). Source data are provided as a Source Data file.

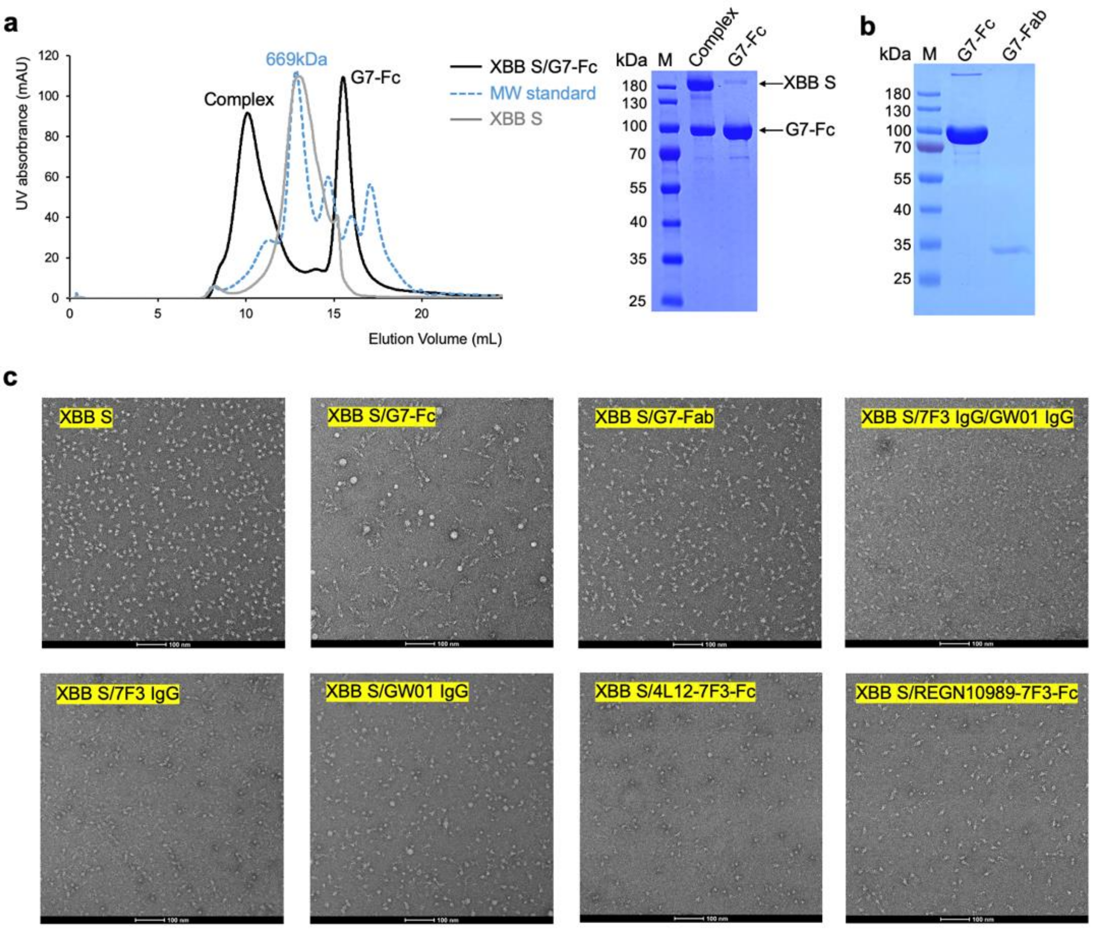

Supplementary Fig. S4. Purification of SARS-CoV-2 XBB S in complex with G7-Fc. a Gel-filtration curve and SDS-PAGE of SARS-CoV-2 XBB S complexed with G7-Fc complex. For comparison, the gel-filtration curves of SARS-CoV-2 XBB S and the molecular weight standards were also shown. **b** SDS-PAGE of G7-Fc and G7-Fab. G7-Fab was obtained by digesting G7-Fc using papain. **c** Negative staining EM images of SARS-CoV-2 XBB S alone, XBB S/G7-Fc complex, XBB S/G7-Fab, XBB S/7F3 IgG/GW01 IgG, XBB S/7F3 IgG, XBB S/GW01 IgG, XBB S/4L12-7F3-Fc and XBB S/REGN10989-7F3-Fc showing that G7-Fc binding induces the formation of trimer dimer. Source data are provided as a Source Data file.

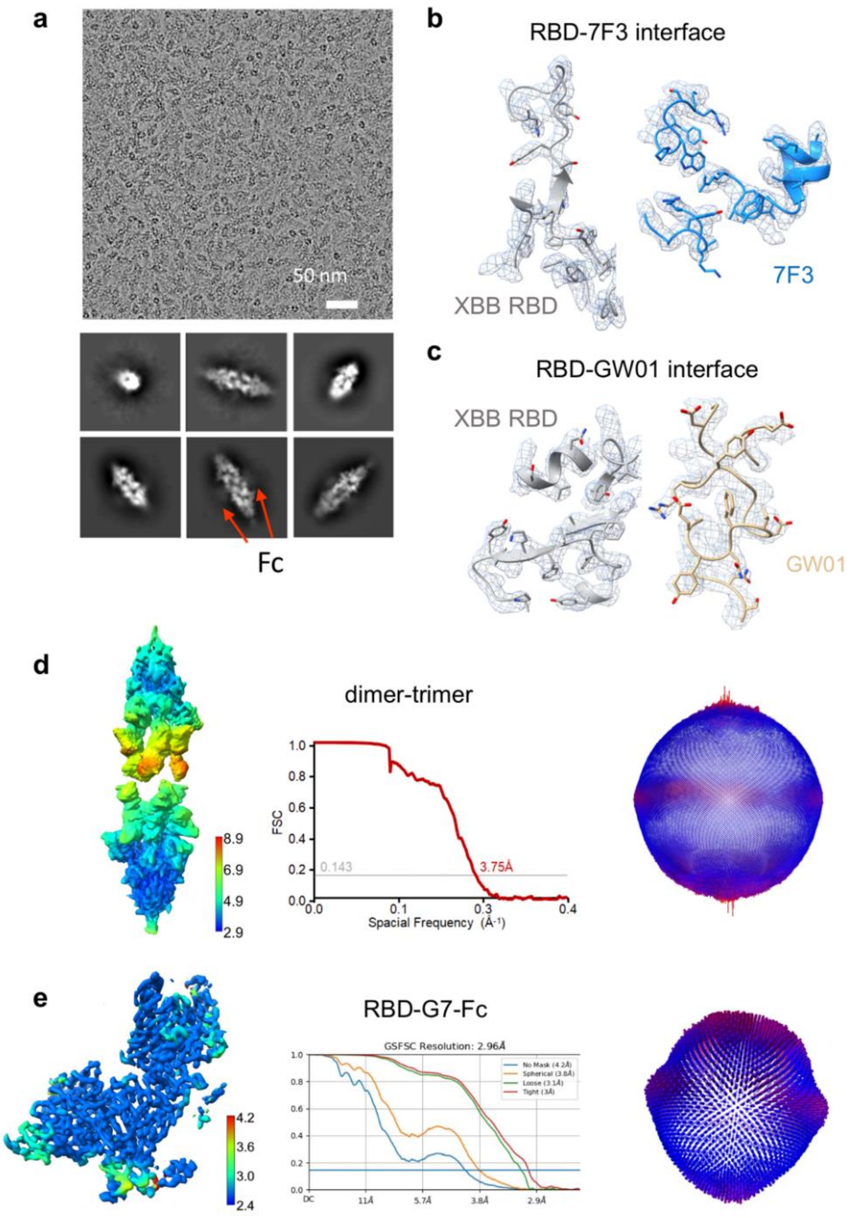

Supplementary Fig. S5. Cryo-EM data collection and processing of SARS-CoV-2 XBB S in complex with G7-Fc. a Representative electron micrograph and 2D classification results of G7-Fc bound XBB S. **b** Electron density maps for secondary structure elements in the RBD-7F3 interface. **c** Electron density maps for secondary structure elements in the RBD-GW01 interface. **d** The reconstruction map of XBB S in complex with G7-Fc and its gold-standard Fourier shell correlation (FSC) curve from cryoSPARC, and angular distribution of particles in final refinement of XBB S in complex with G7-Fc. **e** The local refinment map, FSC curve , and angular distribution of particles in final refinement of RBD-G7-Fc.

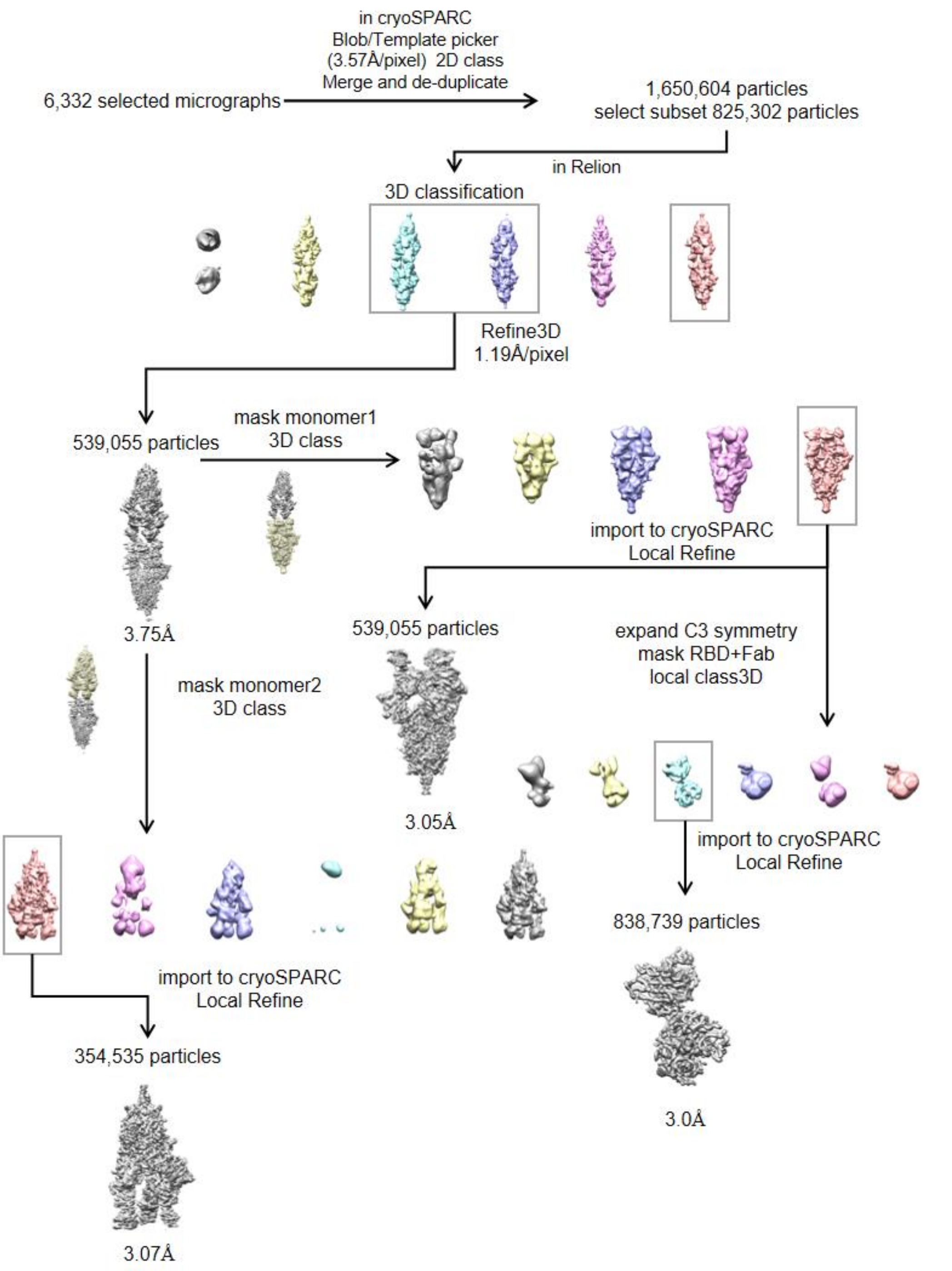

Supplementary Fig. S6. Data processing flowchart of G7-Fc-bound SARS-CoV-2 XBB S trimer.

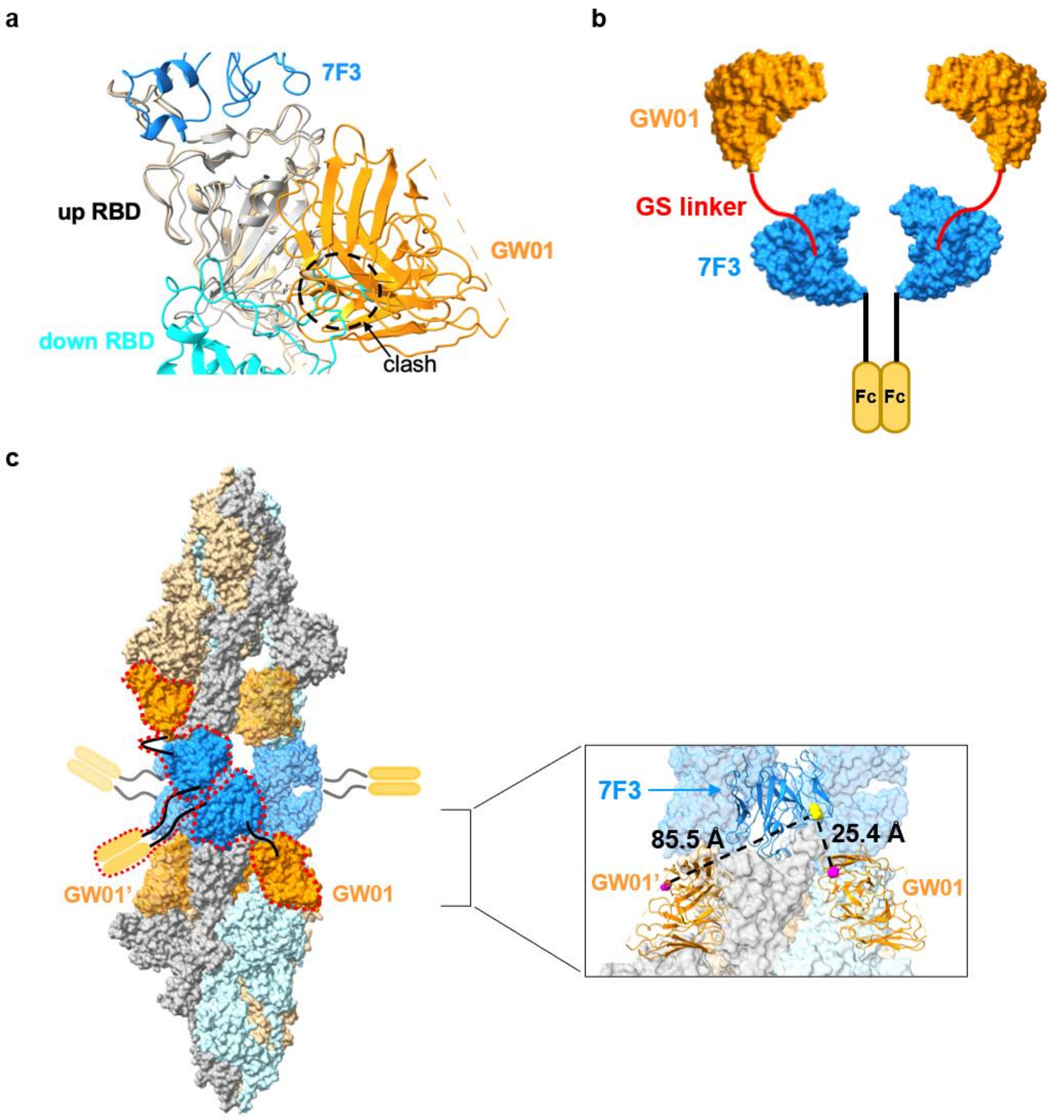

Supplementary Fig. S7. The XBB S/G7-Fc complex formation. a Structure superposition of XBB S-RBD/G7-Fc with apo-state S. Structures are aligned over the up RBD. GW01 clashes with the adjacent down RBD when 7F3 binds with the up RBD. **b** Schematic diagram of G7-Fc. **c** Schematic diagram showing how Fc regions crosslink S trimers. The red dashed line represents a G7-Fc antibody. The zoomed-in view of RBD/G7-Fc is shown on the right panel. Due to the distance limitation of (GGGGS)4 linker (~70Å) between GW01 and 7F3, 7F3 and GW01' bound to the same RBD were derived from two neiboring G7-Fc antibodies. Yellow spheres represent the N-terminal of 7F3 and magenta spheres represent the C-terminal of GW01/GW01'.

a

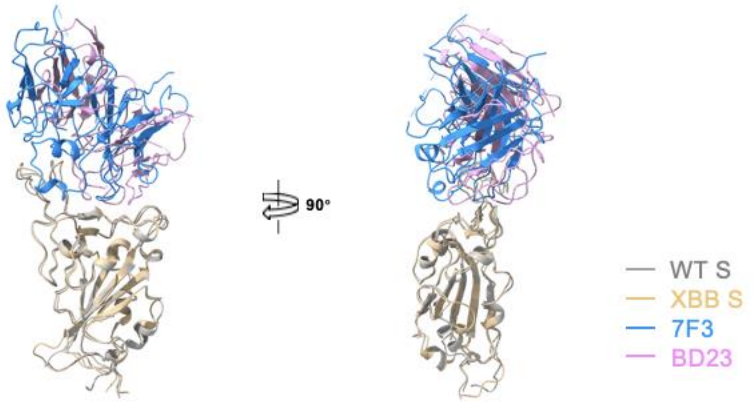

b

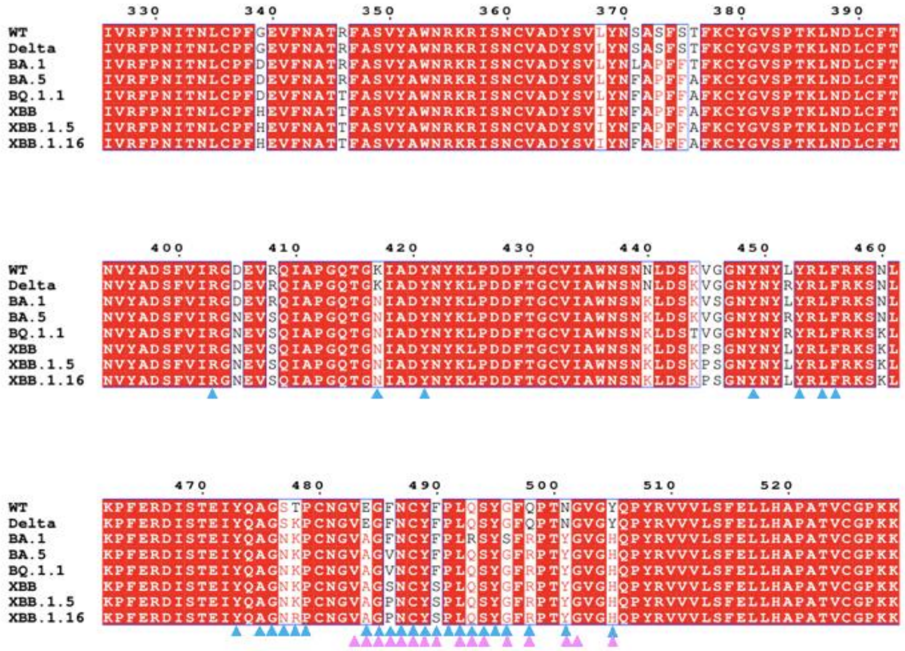

Supplementary Fig. S8. Comparison between 7F3 and BD23. a Structure comparison between XBB S/7F3 and WT S/BD23 (PDB ID: 7BYR). Structures are aligned on RBD. **b** Sequence alignment of SARS-CoV-2 WT, Delta, BA.1, BA.5, BQ.1.1, XBB, XBB.1.5 and XBB.1.16. Residues involved in XBB S/7F3 are marked with triangles in blue. Residues involved in WT S/BD23 are marked with triangles in pink.

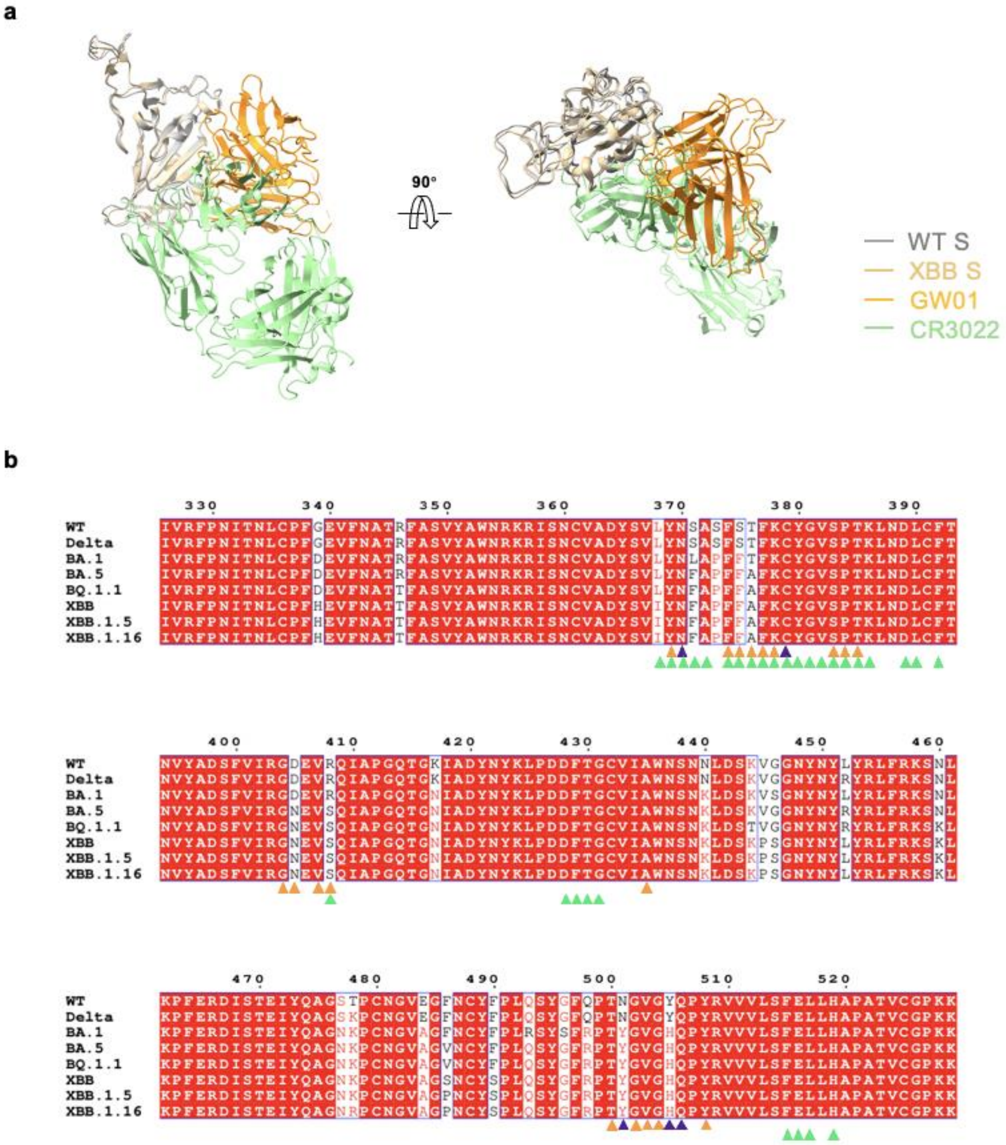

Supplementary Fig. S9. Comparison between GW01 and CR3022. a Structure comparison between XBB S/GW01 and WT S/CR3022 (PDB ID: 6W41). Structures are aligned on RBD. **b** Sequence alignment of SARS-CoV-2 WT, Delta, BA.1, BA.5, BQ.1.1, XBB, XBB.1.5 and XBB.1.16. Residues involved in both XBB S/GW01 and BA.1 S/GW01 are marked with triangles in orange. Other residues involved in BA.1 S/GW01 are marked with triangles in purple. Residues involved in WT S/CR3022 are marked with triangles in green.

a

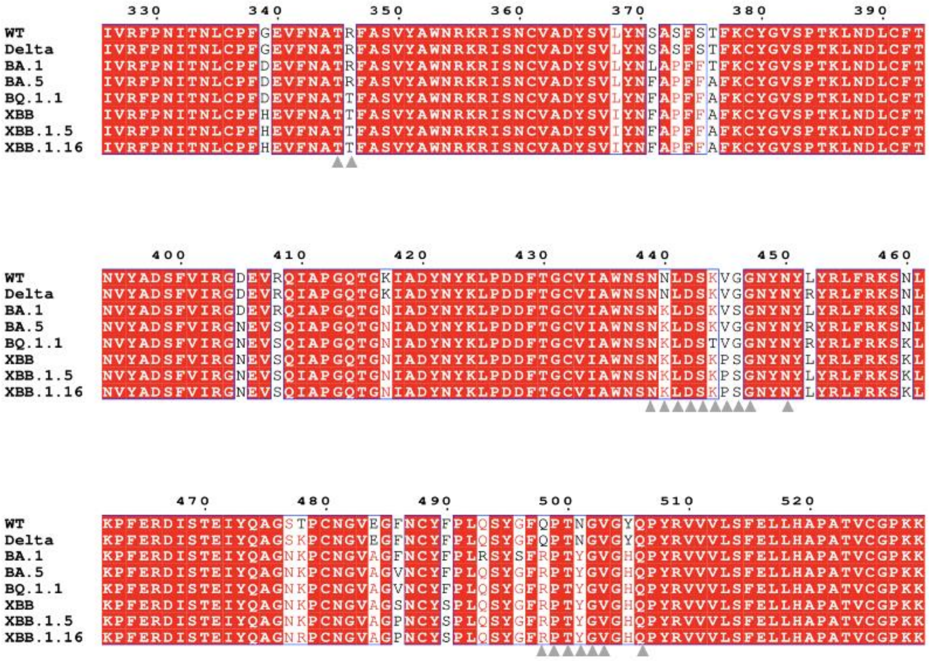

b

Mutants	Sequence No.	Percentage (%)
501Y (XBB)	1099168	95.13
501A	6	0
501C	0	0
501G	1	0
501D	0	0
501E	0	0
501F	8	0
501H	6	0
501I	0	0
501K	0	0
501L	0	0
501M	0	0
501N	2504	0.22
501P	0	0
501Q	0	0
501R	0	0
501S	3	0
501T	2	0
501V	0	0
501W	0	0

Supplementary Fig. S10. Sequence alignment of SARS-CoV-2 WT, Delta, BA.1, BA.5, BQ.1.1, XBB, XBB.1.5 and XBB.1.16. a Conserved amino acids are highlighted in red. Residues involved in LY-CoV1404 are marked with triangles in grey. **b** The mutation rates of other residues in position Y501 in the global SARS-CoV-2 genomic databases since January 2023.

Supplementary Table. S1. Summary of binding affinities of G7-Fc, 7G-Fc, 7F3, GW01, and LY-CoV1404 to the RBD proteins of SARS-CoV-2 subvariants.

Protein	Ab	KD (M)	KD Error	kon(1/Ms)	kon Error	kdis(1/s)	kdis Error
SARS-CoV-2 RBD	G7-Fc	<1.0E-12	<1.0E-12	1.34E+05	3.69E+02	<1.0E-07	/
	7G-Fc	<1.0E-12	<1.0E-12	1.38E+05	1.14E+03	<1.0E-07	/
	7F3	<1.0E-12	3.18E-09	1.92E+05	1.97E+04	<1.0E-07	/
	GW01	4.22E-10	3.74E-11	1.40E+05	3.14E+03	5.89E-05	5.06E-06
	LY-CoV1404	<1.0E-12	<1.0E-12	7.91E+04	3.84E+03	<1.0E-07	/
Delta RBD	G7-Fc	<1.0E-12	<1.0E-12	7.29E+04	2.92E+03	<1.0E-07	/
	7G-Fc	<1.0E-12	<1.0E-12	8.60E+04	4.94E+03	<1.0E-07	/
	7F3	2.08E-08	3.78E-10	4.40E+04	4.75E+02	9.14E-04	1.34E-05
	GW01	<1.0E-12	1.33E-12	1.28E+05	5.12E+03	<1.0E-07	/
	LY-CoV1404	<1.0E-12	6.62E-11	2.44E+05	1.59E+04	<1.0E-07	/
BA.1 RBD	G7-Fc	7.04E-08	2.10E-09	1.06E+04	1.84E+02	7.45E-04	1.81E-05
	7G-Fc	2.60E-06	2.65E-07	5.22E+03	5.60E+01	1.36E-02	1.37E-03
	7F3	N.D.					
	GW01	1.73E-06	1.58E-07	9.55E+03	8.04E+01	1.65E-02	1.50E-03
	LY-CoV1404	<1.0E-12	7.12E-10	3.22E+04	4.30E+03	<1.0E-07	/
BA.5 RBD	G7-Fc	<1.0E-12	<1.0E-12	2.66E+04	2.36E+03	<1.0E-07	/
	7G-Fc	<1.0E-12	<1.0E-12	2.42E+04	2.03E+03	<1.0E-07	/
	7F3	N.D.					
	GW01	7.48E-08	3.83E-09	4.23E+04	1.40E+03	3.17E-03	1.24E-04
	LY-CoV1404	<1.0E-12	<1.0E-12	4.05E+04	3.13E+03	<1.0E-07	/
BF.7 RBD	G7-Fc	<1.0E-12	3.50E-10	3.39E+04	1.50E+03	<1.0E-07	/
	7G-Fc	5.37E-08	7.86E-10	1.55E+04	1.35E+02	8.32E-04	9.80E-06
	7F3	1.28E-06	2.82E-07	3.21E+04	7.89E+02	4.12E-02	9.01E-03
	GW01	3.30E-08	1.11E-09	2.11E+04	4.26E+02	6.97E-04	1.87E-05
	LY-CoV1404	<1.0E-12	2.97E-12	4.68E+04	3.14E+03	<1.0E-07	/
BQ.1.1 RBD	G7-Fc	<1.0E-12	<1.0E-12	8.61E+04	3.87E+03	<1.0E-07	/
	7G-Fc	<1.0E-12	<1.0E-12	1.14E+05	1.17E+04	<1.0E-07	/
	7F3	2.41E-08	3.90E-10	7.98E+04	1.04E+03	1.92E-03	1.83E-05
	GW01	5.62E-07	1.11E-07	1.03E+05	3.60E+03	5.79E-02	1.13E-02
	LY-CoV1404	6.06E-07	4.80E-08	9.03E+04	1.39E+03	5.47E-02	4.25E-03
XBB RBD	G7-Fc	<1.0E-12	4.36E-12	5.31E+04	1.45E+03	<1.0E-07	/
	7G-Fc	9.18E-09	2.86E-10	6.06E+04	1.06E+03	5.56E-04	1.43E-05
	7F3	1.99E-08	1.03E-08	5.66E+04	5.66E+03	1.13E-03	5.72E-04
	GW01	N.D.					
	LY-CoV1404	N.D.					
XBB.1.5 RBD	G7-Fc	<1.0E-12	1.31E-10	4.19E+04	9.36E+02	<1.0E-07	/
	7G-Fc	2.13E-08	4.23E-10	2.49E+04	2.64E+02	5.29E-04	8.87E-06
	7F3	2.58E-09	1.11E-08	3.33E+04	3.42E+04	8.61E-05	3.61E-04
	GW01	1.66E-06	3.17E-07	2.61E+04	6.90E+02	4.34E-02	8.20E-03
	LY-CoV1404	N.D.					
XBB.1.16 RBD	G7-Fc	<1.0E-12	<1.0E-12	9.51E+04	4.02E+03	<1.0E-07	/
	7G-Fc	<1.0E-12	3.64E-10	6.23E+04	6.38E+03	<1.0E-07	/
	7F3	2.86E-08	1.22E-09	1.68E+05	4.61E+03	4.80E-03	1.57E-04
	GW01	<1.0E-12	<1.0E-12	5.51E+04	5.79E+03	<1.0E-07	/
	LY-CoV1404	N.D.					

Supplementary Table. S2. Cryo-EM data collection and refinement statistics.

	Trimer Dimer (EMDB-36423) (PDB 8JMM)	RBD-G7-Fc Local (EMDB-36321) (PDB 8JIN)
**Data collection and processing**		
Magnification	105,000	
Voltage (kV)	300	
Electron exposure (e–/Å^2)	50	
Defocus range (μm)	-1.0 to -3.0	
Pixel size (Å)	1.19	
Symmetry imposed	C1	
Initial particle images (no.)	1,650,604	1,650,604
Final particle images (no.)	539,055	838,739
Map resolution (Å) FSC threshold	0.143	
Map resolution range (Å)	3.75	3.0
**Refinement**		
Initial model used (PDB code)	7WOW	7WOW
Model resolution (Å) FSC threshold	0.143	0.143
Model resolution range (Å)	3.75	3.0
Map sharpening *B* factor (Å^2)	-98.5	-151.1
Model composition		
Non-hydrogen atoms	70,643	5,250
Protein residues	9100	681
Ligands	0	0
B factors (Å^2)		
Protein	41.47	55.42
Ligand	-	-
R.m.s. deviations		
Bond lengths (Å)	0.003	0.002
Bond angles (°)	0.897	0.484
Validation		
MolProbity score	2.13	2.44
Clashscore	6.71	8.09
Poor rotamers (%)	3.83	5.13
Ramachandran plot		
Favored (%)	95.39	92.70
Allowed (%)	4.61	7.15
Disallowed (%)	0.00	0.15

Supplementary Table S3. Interface area (Å2) between RBD and G7-Fc.

Structure 1	Structure 2	Interface area, Å^2
XBB S-RBD	7F3	976.4
XBB S-RBD	GW01	626.6
BA.1 S-RBD	GW01	718.3

Supplementary Table S4. Hydrogen bonds between XBB S-RBD and 7F3(< 4 Å).

##	XBB S-RBD	Dist. [Å]	7F3
1	LEU 455 [N]	3.76	TYR 229 [OH]
2	PHE 456 [N]	3.56	TYR 229 [OH]
3	PHE 456 [O]	3.64	TYR 229 [OH]
4	TYR 473 [OH]	3.01	THR 233 [OG1]
5	LYS 478 [NZ]	2.47	SER 31 [OG]
6	GLY 485 [O]	2.88	ARG 225 [NH1]
7	ASN 487 [ND2]	2.63	TYR 242 [OH]
8	CYS 488 [O]	2.92	ARG 225 [NH1]
9	TYR 489 [OH]	3.87	TYR 226 [N]
10	SER 490 [O]	2.71	TYR 179 [OH]
11	PRO 491 [O]	2.88	TYR 229 [OH]
12	GLN 493 [NE2]	3.25	TYR 179 [OH]
13	GLN 493 [NE2]	2.72	ALA 227 [O]
14	SER 494 [OG]	2.95	LYS 177 [O]
15	SER 494 [N]	2.65	ALA 178 [O]
16	SER 494 [OG]	2.40	ALA 178 [O]
17	SER 494 [OG]	3.67	GLY 180 [N]
18	TYR 501 [OH]	3.82	LYS 177 [NZ]

No disulfide bonds found
No covalent bonds found
No salt bridges found

Supplementary Table S5. Hydrogen bonds between XBB S-RBD and GW01 (< 4 Å).

##	XBB S-RBD	Dist. [Å]	GW01
1	PHE 375 [O]	3.65	ASN 233 [N]
2	PHE 375 [O]	3.32	TYR 234 [OD1]
3	PHE 375 [O]	3.88	GLU 235 [N]
4	PHE 377 [O]	2.50	ASN 233 [ND2]
5	PHE 377 [N]	2.82	ASN 233 [OD1]
6	ASN 405 [OD1]	2.51	TYR 226 [OH]

No disulfide bonds found
No covalent bonds found
No salt bridges found

Supplementary Table S6. Conservation of the hydrogen bonding sites between XBB S-RBD and G7-Fc.

G7-Fc interaction	XBB S-RBD	Conservation (%)
7F3	LEU 455	83.03
	PHE 456	72.13
	TYR 473	95.44
	LYS 478	81.1
	GLY 485	94.9
	ASN 487	95.68
	CYS 488	95.74
	TYR 489	95.66
	SER 490	68.84
	PRO 491	95.67
	GLN 493	95.61
	SER 494	95.03
	TYR 501	94.9
GW01	PHE 375	91.79
	PHE 377	92.59
	LYS 378	92.62
	ASN 405	93.29

Supplementary Table S7. Mutations for BA.2.75，BA.5 and XBB sub-lineage.

Pango lineages	Mutations in spike compared to variants
BL.1	BA.2.75+R346T
BA.2.75.2	BA.2.75+R346T+F486S
BN.2.1	BA.2.75+R346T+F490S
CA.1	BA.2.75+R346T+L452R+F486S
BA.2.75.4	BA.2.75+L452R
BR.1	BA.2.75+L452R+K444M
CH1.1	BA.2.75+K444T+L452R
XBB.1.5	XBB+S486P
XBB.1.16	XBB+S486P+K478R
BF.7	BA.5+R346T
BA.4.6	BA.5+R346T+N658S
BA.5.1.12	BA.5+V445A
BA.5.6.2	BA.5+K444T

Supplementary Table S8. Primers designed for site-directed mutagenesis to construct the BA.2.75, BA.5, and XBB sub-lineages.

site-directed mutagenesis primers	Sequence
R346T-Forward	5'-gtgttcaacgcaaccacgttcgcaagcgtgtac-3'
R346T-Reverse	5'-gtacacgcttgcgaacgtggttgcgttgaacac-3'
K444T-Forward	5'-aacaagctggacagcacggtgggcggcaacta-3'
K444T-Reverse	5'-tagttgccgcccaccgtgctgtccagcttgtt-3'
K444M-Forward	5'-agcaacaagctggattccatggtgagcggcaactacaatt-3'
K444M-Reverse	5'-aattgtagttgccgctcaccatggaatccagcttgttgct-3'
V445A-Forward	5'-aagctggacagcaaggcgggcggcaactacaac-3'
V445A-Reverse	5'-gttgtagttgccgcccgccttgctgtccagctt-3'
L452R-Forward	5'-gggcggcaactacaattatcggtaccggctgttta -3'
L452R-Reverse	5'-taaacagccggtaccgataattgtagttgccgccc -3'
K478R-Forward	5'-ctaccaggcaggcaacaggccatgcaatgga-3'
K478R-Reverse	5'-tccattgcatggcctgttgcctgcctggtag-3'
F486S-Forward	5'-atgcaatggagtggcgggcagtaactgttatttccctctg-3'
F486S-Reverse	5'-cagagggaaataacagttactgcccgccactccattgcat-3'
S486P-Forward	5'-caatggagtggcgggccctaactgttattctcct-3'
S486P-Reverse	5'-aggagaataacagttagggcccgccactccattg-3'
F490S-Forward	5'-agtggcgggctttaactgttatagccctctgcagag-3'
F490S-Reverse	5'-ctctgcagagggctataacagttaaagcccgccact-3'
N658S-Forward	5'-cgccgaatatgtgaacagcagctacgagtgcgac-3'
N658S-Reverse	5'-gtcgcactcgtagctgctgttcacatattcggcg-3'
