# Supplementary material for: A bispecific antibody exhibits broad neutralization against SARS-CoV-2 Omicron variants XBB.1.16, BQ.1.1 and sarbecoviruses
Source: Nat Commun. 2024 Jun 15;15:5127. doi: 10.1038/s41467-024-49096-1 (PMC11180174; doi:10.1038/s41467-024-49096-1)
Supplement: Supplementary file 3 — Reporting Summary [file 41467_2024_49096_MOESM3_ESM.pdf]

nature portfolio

Corresponding author(s): Yingdan Wang, Aihua Hao, Ping Ji, Yunping Ma, Zhaoyong Zhang, Jiali Chen, Qiyu Mao, Xinyi Xiong, Palizhati Rehati, Yajie Wang,Yanqun Wang, Yumei Wen, Lu Lu, Zhenguo Chen, Jincun Zhao, Fan Wu, Jinghe Huang, Lei Sun

Last updated by author(s): May 12, 2024

Reporting Summary

Nature Portfolio wishes to improve the reproducibility of the work that we publish. This form provides structure for consistency and transparency in reporting. For further information on Nature Portfolio policies, see our Editorial Policies and the Editorial Policy Checklist.

Statistics

For all statistical analyses, confirm that the following items are present in the figure legend, table legend, main text, or Methods section.

n/a	Confirmed	
☐	☒	The exact sample size (*n*) for each experimental group/condition, given as a discrete number and unit of measurement
☐	☒	A statement on whether measurements were taken from distinct samples or whether the same sample was measured repeatedly
☐	☒	The statistical test(s) used AND whether they are one- or two-sided<br>*Only common tests should be described solely by name; describe more complex techniques in the Methods section.*
☒	☐	A description of all covariates tested
☒	☐	A description of any assumptions or corrections, such as tests of normality and adjustment for multiple comparisons
☐	☒	A full description of the statistical parameters including central tendency (e.g. means) or other basic estimates (e.g. regression coefficient) AND variation (e.g. standard deviation) or associated estimates of uncertainty (e.g. confidence intervals)
☐	☒	For null hypothesis testing, the test statistic (e.g. *F*, *t*, *r*) with confidence intervals, effect sizes, degrees of freedom and *P* value noted<br>*Give P values as exact values whenever suitable.*
☒	☐	For Bayesian analysis, information on the choice of priors and Markov chain Monte Carlo settings
☒	☐	For hierarchical and complex designs, identification of the appropriate level for tests and full reporting of outcomes
☒	☐	Estimates of effect sizes (e.g. Cohen's *d*, Pearson's *r*), indicating how they were calculated

Our web collection on statistics for biologists contains articles on many of the points above.

Software and code

Policy information about availability of computer code

Data collection	All software and algorithms used in this study are open source.<br>Cryo-EM data collection: Cryo-EM data were captured on a TITAN Krios G4 TEM (Thermo Fisher Scientific) equipped with a Falcon 4i camera and a Selectris X Imaging filter (Thermo Fisher Scientific). Automated data collection was performed with EPU software in AFIS mode.<br>All of flow cytometry data were collected by BD BD FACSAria II. Chemiluminescence signals data were detected by PerkinElmer EnSight. Absorbance Absorb data were collected by BioTek Epoch 2. All of bilayer interferometry data were collected by Octet RED96.
Data analysis	All software and algorithms used in this study are open source.<br>Cryo-EM data analysis:<br>Movie stacks were imported to Relion3.1, then motion corrected by MotionCor2 and CTF estimated by Gctf. 3D reconstruction was performed using Relion3.1 and cryoSPARC v4.0.3. Maps were sharpened using DeepEMhancer, and handedness was corrected using UCSF Chimera.<br>Model building and refinement:<br>The initial model was generated using swiss-model, fitted into the maps using UCSF Chimera and then manually adjusted using COOT. Real space refinement were performed using PHENIX. Model validation was performed using Molprobity. Figures were prepared using UCSF Chimera and UCSF ChimeraX.<br>Flow cytometry data were analyzed by FlowJo_v10. Data of ELISA and neutralization assay is analyzed by GraphPad Prism9.5. Data of BLI is analyzed FortéBio Data Analysis 8.1.

For manuscripts utilizing custom algorithms or software that are central to the research but not yet described in published literature, software must be made available to editors and reviewers. We strongly encourage code deposition in a community repository (e.g. GitHub). See the Nature Portfolio guidelines for submitting code & software for further information.

Data

Policy information about availability of data

All manuscripts must include a data availability statement. This statement should provide the following information, where applicable:

- Accession codes, unique identifiers, or web links for publicly available datasets
- A description of any restrictions on data availability
- For clinical datasets or third party data, please ensure that the statement adheres to our policy

Coordinates and maps associated with data reported in this manuscript are deposited to the Electron Microscopy Data Bank (EMDB) and Protein Data Bank (PDB) with accession numbers EMD-36423 and PDB 8JMM (XBB S trimer-dimer/G7-Fc) , EMD-36321 and PDB 8JIN (local refined map of XBB S/G7-Fc). Accession number of GW01 heavy chain and light chain are OP480801.1 and OP480802.1 in GenBank.

Research involving human participants, their data, or biological material

Policy information about studies with human participants or human data. See also policy information about sex, gender (identity/presentation), and sexual orientation and race, ethnicity and racism.

Reporting on sex and gender	This study involved only one human patient, who is male.
Reporting on race, ethnicity, or other socially relevant groupings	The participant didn't belong to any socially relevant grouping as far as we know.
Population characteristics	The human involved in this study was a 40-year-old male, who is recovered from COVID-19. Blood sample was collected on the day of discharge.
Recruitment	The serum of the patient demonstrated robust neutralizing activity against SARS-CoV-2 at the time of discharge. Therefore his PBMCs were used for antibody isolation.
Ethics oversight	Ethical approval about the human participant was obtained from the Ethics Committee of the Shanghai Public Health Clinical Center for this study (YJ-2020-S021-01).

Note that full information on the approval of the study protocol must also be provided in the manuscript.

Field-specific reporting

Please select the one below that is the best fit for your research. If you are not sure, read the appropriate sections before making your selection.

[x] Life sciences   [ ] Behavioural & social sciences   [ ] Ecological, evolutionary & environmental sciences

For a reference copy of the document with all sections, see nature.com/documents/nr-reporting-summary-flat.pdf

Life sciences study design

All studies must disclose on these points even when the disclosure is negative.

Sample size	There were 45 mice used in the study. Twenty-five K18-ACE2 mice and twenty BALB/c mice were divided into five groups, respectively. T he number of in each group is five and four, respectively, which meet the requirement of statistical analysis.
Data exclusions	No data were excluded.
Replication	The prophylactic and therapeutic experiment were done in both BALB/c and K18-ACE2 mice. Results in both experiments were mutual corroborate, which indicated that the result are reproducible.
Randomization	Mice were divided into 5 group randomly.
Blinding	The investigator were not blinded to different group.

Reporting for specific materials, systems and methods

We require information from authors about some types of materials, experimental systems and methods used in many studies. Here, indicate whether each material, system or method listed is relevant to your study. If you are not sure if a list item applies to your research, read the appropriate section before selecting a response.

Materials & experimental systems

n/a	Involved in the study	
	x	Antibodies
	x	Eukaryotic cell lines
x		Palaeontology and archaeology
	x	Animals and other organisms
x		Clinical data
x		Dual use research of concern
x		Plants

Methods

n/a	Involved in the study	
x		ChIP-seq
	x	Flow cytometry
x		MRI-based neuroimaging

Antibodies

Antibodies used

PE-Cy™7 Mouse Anti-Human CD19, BD Pharmingen™, Cat No:341093, Clone No. SJ25C1.
FITC Mouse Anti-Human IgD. BD Pharmingen™, Cat No. 555778, Clone No. IA6-2.
PE-Anti-Human IgM, Jackson ImmunoResearch, Code No. 709-116-073.
APC-anti-Human IgA, Jackson ImmunoResearch, Code No. 109-135-011.
Peroxidase AffiniPure™ Goat Anti-Human IgG, Jackson ImmunoResearch, Code No.109-035-098, Lot: 164726

Validation

We used the antibodies following the manufacture instruction. All antibodies performed well.

PE-Cy™7 Mouse Anti-Human CD19,(BD Pharmingen™, Mouse BALB/c IgG1, κ, also know as B4 or B-lymphocyte antigen CD19, specific for human, application for Flow cytometry, 5 µL Per Test)
https://www.bdbiosciences.com/en-us/products/reagents/flow-cytometry-reagents/clinical-discovery-research/single-color-antibodies-ruo-gmp/pe-cy-7-mouse-anti-human-cd19.341093

FITC Mouse Anti-Human IgD （BD Pharmingen™, Reactivity to human (QC Testing), Mouse BALB/c IgG2a, κ, application: for Flow cytometry (Routinely Tested), 20 µl Per Test)
https://www.bdbiosciences.com/en-us/products/reagents/flow-cytometry-reagents/research-reagents/single-color-antibodies-ruo/fitc-mouse-anti-human-igd.555778

PE-Anti-Human IgM (Jackson ImmunoResearch, Target human, Donkey $F(ab')_2$ Fragment, IgM, Fc5µ fragment specific, Minimal Cross Reactivity to Bovine, Horse Serum Proteins), 1:100 dilution for usage.
https://www.jacksonimmuno.com/catalog/products/709-116-073

APC-anti-Human IgA (Jackson ImmunoResearch, Target human, Host: Goat whole IgG, Serum IgA, α chain specific, Conjugate: Allophycocyanin (APC)), 1:100 dilution for usage.
https://www.jacksonimmuno.com/catalog/products/109-135-011

Peroxidase AffiniPure™ Goat Anti-Human IgG (Jackson ImmunoResearch, Target human, Goat whole IgG, gG, Fcγ fragment specific, Minimal Cross reactivity to Bovine, Horse, Mouse Serum Proteins),1:2500 dilution for usage.
https://www.jacksonimmuno.com/catalog/products/109-035-098

Eukaryotic cell lines

Policy information about cell lines and Sex and Gender in Research

Cell line source(s)

HEK 293T cell, ATCC, CRL-3216
HEK 293F cell,Thermo Fisher Scientific, R79007
Huh7 cell: National Collection of Authenticated Cell Cultures. CSTR: 19375.09.3101HUMSCSP526
3T3CD40L: NIH reagent program

Authentication

None of the cell line were authenticated.

Mycoplasma contamination

Mycoplasma killing reagents were added into the cell culture medium to avoid mycoplasma contamination. PCR was used to confirmed there was no mycoplasma contamination in the cell lines.

Commonly misidentified lines
(See ICLAC register)

The cell lines used in this study are not included in the ICLAC list.

Animals and other research organisms

Policy information about studies involving animals; ARRIVE guidelines recommended for reporting animal research, and Sex and Gender in Research

Laboratory animals	6-week-old BALB/c and K18-ACE2 mice
Wild animals	No wild animal was involved in this study.
Reporting on sex	All of mice involved in this study are female.
Field-collected samples	This study did not involved samples collected from filed.
Ethics oversight	the Institutional Animal Care and Use Committees of Affiliated First Hospital of Guangzhou Medical University (Approval Number: 20230615).

Note that full information on the approval of the study protocol must also be provided in the manuscript.

Plants

Seed stocks	*Report on the source of all seed stocks or other plant material used. If applicable, state the seed stock centre and catalogue number. If plant specimens were collected from the field, describe the collection location, date and sampling procedures.*
Novel plant genotypes	*Describe the methods by which all novel plant genotypes were produced. This includes those generated by transgenic approaches, gene editing, chemical/radiation-based mutagenesis and hybridization. For transgenic lines, describe the transformation method, the number of independent lines analyzed and the generation upon which experiments were performed. For gene-edited lines, describe the editor used, the endogenous sequence targeted for editing, the targeting guide RNA sequence (if applicable) and how the editor was applied.*
Authentication	*Describe any authentication procedures for each seed stock used or novel genotype generated. Describe any experiments used to assess the effect of a mutation and, where applicable, how potential secondary effects (e.g. second site T-DNA insertions, mosiacism, off-target gene editing) were examined.*

Flow Cytometry

Plots

Confirm that:

- [x] The axis labels state the marker and fluorochrome used (e.g. CD4-FITC).
- [x] The axis scales are clearly visible. Include numbers along axes only for bottom left plot of group (a 'group' is an analysis of identical markers).
- [x] All plots are contour plots with outliers or pseudocolor plots.
- [x] A numerical value for number of cells or percentage (with statistics) is provided.

Methodology

Sample preparation	Blood was draw from COVID-19 recovered patient. PBMCs were isolated immediately.
Instrument	BD FACSAria II
Software	All of the flow cytometry data was analyzed by FlowJo_v10.
Cell population abundance	IgG+ memory B cell population within the PBMC population was 0.8%, which is a regular cell population in human.
Gating strategy	Initially, lymphocytes were identified and gated from the overall PBMC. Within the lymphocytes gate, single memory B cells were further identified and gated. Within the single memory B cell gate, further gating was applied to exclude B cells expressing IgA, IgD, and IgM. The IgG-positive memory B cells were selectively chosen by excluding the other Ig isotypes.

- [x] Tick this box to confirm that a figure exemplifying the gating strategy is provided in the Supplementary Information.
